# Inversion of allosteric effect of arginine on N-acetylglutamate synthase, a molecular marker for evolution of tetrapods

**DOI:** 10.1186/1471-2091-9-24

**Published:** 2008-09-18

**Authors:** Nantaporn Haskins, Maria Panglao, Qiuhao Qu, Himani Majumdar, Juan Cabrera-Luque, Hiroki Morizono, Mendel Tuchman, Ljubica Caldovic

**Affiliations:** 1 Research Center for Genetic Medicine, Children's National Medical Center, 111 Michigan Ave NW, Washington DC, 20010, USA; 2 School of Medicine and Health Sciences, The George Washington University, 2300 I St. NW, Washington DC, 20037, USA; 3 Division of Neurosciences, Beckman Research Institute, 1450 E Duarte Road, Duarte, CA 91010, USA

## Abstract

**Background:**

The efficient conversion of ammonia, a potent neurotoxin, into non-toxic metabolites was an essential adaptation that allowed animals to move from the aquatic to terrestrial biosphere. The urea cycle converts ammonia into urea in mammals, amphibians, turtles, snails, worms and many aquatic animals and requires N-acetylglutamate (NAG), an essential allosteric activator of carbamylphosphate synthetase I (CPSI) in mammals and amphibians, and carbamylphosphate synthetase III (CPSIII) in fish and invertebrates. NAG-dependent CPSI and CPSIII catalyze the formation of carbamylphosphate in the first and rate limiting step of ureagenesis. NAG is produced enzymatically by N-acetylglutamate synthase (NAGS), which is also found in bacteria and plants as the first enzyme of arginine biosynthesis. Arginine is an allosteric inhibitor of microbial and plant NAGS, and allosteric activator of mammalian NAGS.

**Results:**

Information from mutagenesis studies of *E. coli *and *P. aeruginosa *NAGS was combined with structural information from the related bacterial N-acetylglutamate kinases to identify four residues in mammalian NAGS that interact with arginine. Substitutions of these four residues were engineered in mouse NAGS and into the vertebrate-like N-acetylglutamate synthase-kinase (NAGS-K) of *Xanthomonas campestris*, which is inhibited by arginine. All mutations resulted in arginine losing the ability to activate mouse NAGS, and inhibit *X. campestris *NAGS-K. To examine at what point in evolution inversion of arginine effect on NAGS occur, we cloned NAGS from fish and frogs and examined the arginine response of their corresponding proteins. Fish NAGS were partially inhibited by arginine and frog NAGS were activated by arginine.

**Conclusion:**

Difference in arginine effect on bacterial and mammalian NAGS most likely stems from the difference in the type of conformational change triggered by arginine binding to these proteins. The change from arginine inhibition of NAGS to activation was gradual, from complete inhibition of bacterial NAGS, to partial inhibition of fish NAGS, to activation of frog and mammalian NAGS. This change also coincided with the conquest of land by amphibians and mammals.

## Background

**Figure 1:**
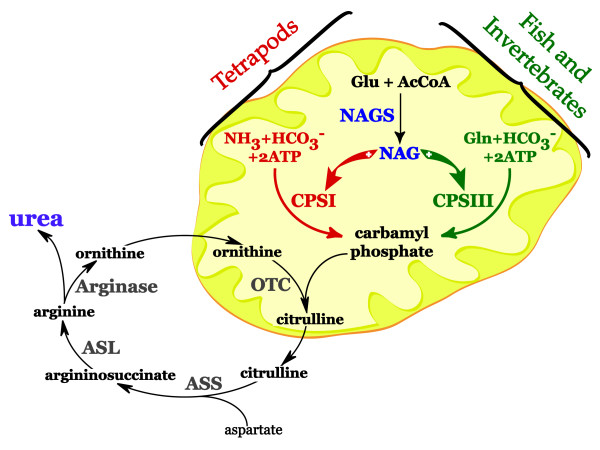
**Urea cycle in tetrapods, fish and invertebrates**. The first three enzymes of the urea cycle are localized in the mitochondria; the remaining three enzymes are cytoplasmic. Mammals and amphibians (tetrapods) have CPSI, which catalyzes the formation of CP from ammonia, bicarbonate and ATP. NAG is an essential allosteric activator of CPSI. CPSIII catalyzes the formation of CP from glutamine, bicarbonate and ATP in fish and invertebrates. The enzymatic activity of CPSIII increases in the presence of NAG. Abbreviations: NAGS – N-acetylglutamate synthase; NAG – N-acetylglutamate; CPSI – carbamylphosphate synthetase I; CPSIII – carbamylphosphate synthetase III; OTC – ornithine transcarbamylase; ASS – argininosuccinate synthase; ASL – argininosuccinate lyase.

The efficient incorporation of ammonia nitrogen into compounds that can be easily excreted was essential for animals to move from the aquatic biosphere to land [1,2]. Ammonia is a waste product of nitrogen metabolism and a potent neurotoxin. In mammals, blood ammonia levels are maintained in the low micromolar range (< 50 μM), as compared to other metabolites, such as lactate or ketone bodies, that are tolerated in concentrations that are orders of magnitude higher (1–2 mM) [3]. The threshold of tolerance for elevated blood ammonia levels is very low and concentrations above 100 μM can cause signs of brain dysfunction, and even higher levels can lead to coma and death [3]. Therefore, the main function of ammonia disposal systems in animals is to protect their central nervous systems from the harmful effects of ammonia [3,4]. The direct excretion of ammonia, which at physiological pH is predominantly in the form of the ammonium ion, requires large quantities of water. Aquatic animals can excrete ammonia directly into their environment due to the large concentration gradient of ammonia between their plasma and surrounding water [2]. However, the efficient disposal of ammonia in a manner that does not require as much water was essential for life on land and adaptation of aquatic animals to the terrestrial biosphere [5]. Effective ammonia disposal is also essential for life in extreme aquatic environments such as high pH [6,7], or upon occasional exposure to air during drought [8-11] and low tides [12,13]. Two metabolic pathways, the urea cycle and urate pathway function in the liver to efficiently convert ammonia into urea and uric acid, which are non-toxic and easily excreted [14]. Based on the type of predominant nitrogen waste product, ammonia, urea or urate, animals have been classified as ammonotelic, ureotelic and uricotelic [15]. However, this division is not strict because many animals, such as snails, turtles and fish, excrete more than one nitrogen waste product, and the preference for specific nitrogen waste products changes as the environment of the animal changes [8-11,16-24]. In mammals, amphibians, gastropods and some turtles ammonia is detoxified through ureagenesis (Figure 1) [3,14,16-18,20,25-33], while lizards, snakes, birds, insects and terrestrial crustaceans use uric acid to eliminate nitrogen waste [34-36]. Many aquatic animals that are capable of ureagenesis do not use urea as the major nitrogen waste product [12,13,21,29,37-43]. In sharks, skates and rays, urea functions as an osmolyte [38,44-50] but its function in bony fishes is not well understood [2,39,51,52].

The first reaction of urea cycle is formation of carbamylphosphate (CP, Figure 1). In tetrapods, carbamylphosphate synthetase I (CPSI, EC 6.3.4.16) produces CP from ammonia, bicarbonate and ATP [3]. In fish and invertebrates, the formation of CP is catalyzed by carbamylphosphate synthetase III (CPSIII; EC 6.3.3.5), which uses glutamine, bicarbonate and ATP as substrates [53]. N-acetylglutamate (NAG), which is formed enzymatically by N-acetylglutamate synthase (NAGS; EC 2.3.1.1) from glutamate and acetyl coenzyme A (Figure 1), is an essential allosteric activator of CPSI, and deficiency of NAG results in a block in ureagenesis [4,54]. NAG also activates CPSIII, but the effect of NAG on the enzymatic activity of CPSIII is more complex. At high glutamine concentrations, CPSIII is active even in the absence of NAG, while at low glutamine concentrations, CPSIII requires NAG for activity [41,44,55]. Some fish CPSIII have been shown to also be capable of using ammonia as substrate, and in these enzymes, NAG is required for the formation of CP from ammonia, bicarbonate and ATP [41,44,55]. Sequence comparisons and mutagenesis studies suggest that CPSI evolved from CPSIII as a consequence of losing the ability to bind glutamine [56,57].

The urea cycle and the microbial arginine biosynthesis pathway have six enzymes and intermediates in common, leading to the hypothesis that the urea cycle evolved from the arginine biosynthesis pathway [4,58]. Indeed, sequences of all six enzymes of the urea cycle are similar across phyla [59-62]. However, among these enzymes, NAGS is the most diverse [63]. Phylogenetic analysis of NAGS proteins indicated that they belong to two distinct families. Bacterial and plant NAGS with sequences similar to *Escherichia coli *NAGS are in one family, while the bifunctional N-acetylglutamate synthase and kinase (NAGS-K) from *Xanthomonas campestris*, together with putative NAGS-K from several orders of gamma- and alpha-proteobacteria, vertebrate and fungal NAGS, and fungal N-acetylglutamate kinase belong to a family of vertebrate-like NAGS [62]. Sequence and structural alignments of vertebrate-like NAGS sequences reveal four regions in these proteins. At the N-terminus of eukaryotic NAGS is a mitochondrial targeting sequence [64]. Following it is a variable segment, a sequence that is poorly conserved in vertebrate NAGS [65]. The remainder of the protein ("conserved segment") is conserved in vertebrates, fungi and bacteria and it consists of N-terminal kinase and C-terminal acetyltransferase domains [62,66]. The three-dimensional structure of NAGS as well as mutations in patients with NAGS deficiency indicate that the active site of the NAGS is localized in the acetyltransferase domain [54,66], while mutagenesis studies in NAGS from *Pseudomonas aeruginosa*, which is similar to *E. coli *NAGS, indicate that the arginine binding site is localized in the kinase domain [67]. Microbial and plant NAGS are inhibited by arginine, as part of feedback regulation of arginine biosynthesis [58]. Mammalian NAGS is activated by arginine [68-71].

The goals of this study were to examine if differences in arginine binding could explain inversion of the allosteric effect of arginine on NAGS, and determine when during evolution this inversion occurred. The results presented herein imply that binding of arginine to a site conserved in all vertebrate-like NAGS (bacterial and mammalian) induces conformational changes, which exert an opposite effect on enzymatic activity of bacterial and mammalian NAGS. Furthermore, the results show that the allosteric inversion of the effect of arginine occurred during evolution of amphibians and coincided with the conquest of land by ureotelic tetrapods.

## Results

### Arginine Binding Site is Conserved in Mammalian and Bacterial NAGS

**Figure 2:**
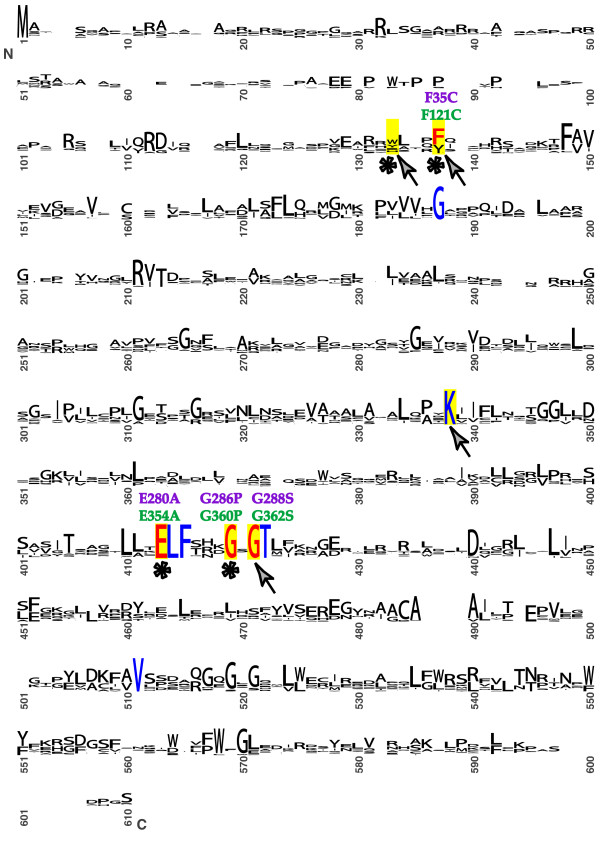
**Conservation of amino acid sequences of NAGS from 25 organisms.** The sizes of letters indicate the degree of conservation. Residues that are important for arginine binding are highlighted in yellow. Invariant residues are shown in blue. Asterisks indicate invariant residues that are mutated in arginine-insensitive NAGS from *E. coli*. Arrows indicate conserved amino acids that are absent or replaced by other amino acids in *E. coli *NAGK, which is not inhibited by arginine. Amino acids that were mutated in this study are shown in red; mutations in the mouse NAGS are shown in green; mutations in the *X. campestris *NAGS-K are in purple. LOGO-alignment was generated using NAGS sequences from five mammals (human, mouse, rat, dog and cow), two amphibians (*X. laevis *and *X. tropicalis*), zebrafish, pufferfish, freshwater pufferfish, arabidopsis, soy, tomato, rice, corn and 11 bacteria (*E. coli*, *R. eutropha*, *N. gonorrhoeae*, *P aeruginosa*, *P. syringiae*, *X. campestris*, *X. axonopodis*, *X. fastidiosa*, *P. bermudensis*, *O. alexandrii *and *M. maris*), which were aligned using ClustalW.

We identified amino acids responsible for binding of arginine to vertebrate-like NAGS based on the information from three dimensional structure of *Thermotoga maritima *N-acetylglutamate kinase (NAGK) protein liganded with L-arginine [72] and mutagenesis studies in NAGS from *P. aeruginosa *and *E. coli *[67,73]. Figure 2 shows an alignment of NAGS proteins from 25 species of bacteria, plants and vertebrates. Nine amino acids are invariant in NAGS, eight of them are located in the kinase domain of NAGS and also conserved in *T. maritima *NAGK [62] (Figure 2). Four of the invariant residues are important for arginine binding; the corresponding residues of *T. maritima *NAGK are in direct contact with arginine [72]. In addition to the four invariant residues, two more amino acids in the region corresponding to the N-helix of *T. maritima *NAGK [72] are important for arginine binding; large, aromatic residues are found in both positions in all NAGS that were examined in this study. The four conserved residues important for arginine binding were also found previously by our group to be mutated in arginine insensitive NAGS from *E. coli *[73]; they are labeled with asterisks in Figure 2. When three of the residues important for binding of arginine were replaced with alanine in NAGS from *P. aeruginosa*, an *E. coli*-like enzyme, it also became insensitive to inhibition by arginine [67]. Figure 2 also shows four amino acids (arrows), which are conserved in NAGS and arginine-inhibited *T. maritima *NAGK, and replaced by other residues or absent in *E. coli *NAGK, which is not subject to arginine inhibition [74].

**Figure 3:**
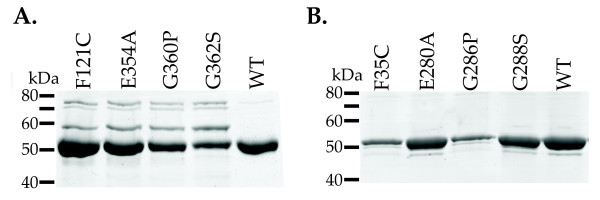
**Purification of wild-type and arginine-insensitive NAGS.** Wild-type and mutant mouse NAGS (A) and *X. campestris *NAGS-K (B) were overexpressed in *E. coli *and purified using nickel-affinity chromatography.

We engineered four amino acid substitutions in the mouse NAGS (mNAGS) and in the vertebrate-like NAGS-K from *Xanthomonas campestris *(XcNAGS-K). The E354A and G360P amino acid substitutions in mNAGS, and E280A and G286P replacements in XcNAGS-K were chosen based on the corresponding sequence changes in *E. coli *NAGK, which is not inhibited by arginine [74]. The G362S and F121C amino acid replacements in mNAGS and the corresponding G288S and F35C mutations in XcNAGS-K were among arginine resistant mutants of *E. coli *NAGS [73]. After site directed mutagenesis, four mutant and wild-type mNAGS proteins, as well as mutant and wild-type XcNAGS-K were overexpressed in *E. coli *and purified using nickel-affinity chromatography. Figure 3 shows purification results for mutant and wild-type mNAGS (Panel A) and XcNAGS-K (Panel B). The yields of purified proteins differed, possibly due to differences in stability of mutant proteins compared to wild-type enzymes.

Table 1 shows that in mouse NAGS, all four mutations abolish arginine activation. The G362S mutant had lower specific activity than wild-type NAGS, possibly due to an additional effect of the replacement of a small glycine residue with bulky, polar serine on protein function and/or stability. In the mutant XcNAGS-K enzymes, the effect of arginine was tested on both the synthase and kinase activities. The synthase activity of all four mutant XcNAGS-K proteins was lower than the specific activity of wild-type enzyme (Table 2). The E280A mutation completely abolished inhibition of XcNAGS-K synthase and kinase activities by arginine, while both enzymatic activities of the F35C mutant XcNAGS-K were only partially inhibited by 1 mM arginine. The synthase activities of the G286P and G288S mutants were not inhibited by arginine, and the kinase activity of these two XcNAGS-K mutants decreased in the presence of arginine by 33% and 25%, respectively. It is possible that G286P and G288S mutations severely diminish, but not abolish, arginine binding to XcNAGS-K. Therefore, in these two mutants, arginine may not be able to affect the remote synthase catalytic site, but could still have a weak effect on the kinase active site, which is in close proximity to the arginine binding site [66,72]. These results document elimination of arginine effect, caused by the same mutations in both arginine activated and arginine inhibited NAGS, strongly suggesting that the arginine binding sites in these proteins are conserved. Thus, differences in the conformational dynamics of arginine activated and arginine inhibited NAGS proteins must lead to different allosteric effects of arginine on bacterial and mammalian NAGS.

**Table 1:** Effects of L-arginine on the enzymatic activity of arginine-resistant and wild-type mouse NAGS.

	**Specific Activity ** (μmol min^-1^mg^-1^)	
**Mouse NAGS**		**+1 mM Arginine**

**Wild-type**	12.27 ± 0.19^a^	25.56 ± 0.43
**F121C**	12.22 ± 0.15	13.08 ± 0.07
**E354A**	15.47 ± 0.22	15.28 ± 0.33
**G360P**	13.00 ± 0.12	12.57 ± 0.38
**G362S**	3.22 ± 0.01	3.19 ± 0.09

^a^Activities are averages of three measurements and their associated standard errors

**Table 2:** Effects of L-arginine on synthase and kinase activities of the arginine resistant and wild-type XcNAGS-K.

	**Synthase Activity ** (μmol min^-1^mg^-1^)		**Kinase Activity ** (μmol min^-1^mg^-1^)	
	
**XcNAGS-K**		**+1 mM Arginine**		**+1 mM Arginine**
**Wild-type**	71.00 ± 1.07^a^	nd^b^	61.93 ± 0.83	7.52 ± 1.26
**F35C**	9.66 ± 0.25	4.31 ± 0.01	65.14 ± 4.77	44.35 ± 2.70
**E280A**	26.93 ± 0.68	26.68 ± 0.50	82.13 ± 1.79	82.44 ± 0.16
**G286P**	3.51 ± 0.04	3.39 ± 0.08	76.57 ± 3.97	50.70 ± 7.43
**G288S**	46.04 ± 1.09	42.55 ± 1.02	55.62 ± 1.26	42.92 ± 5.42

^a^Activities are averages of three measurements and their associated standard errors

^b^Not detectable.

### Survey of Urea Cycle Genes in Animals

**Figure 4:**
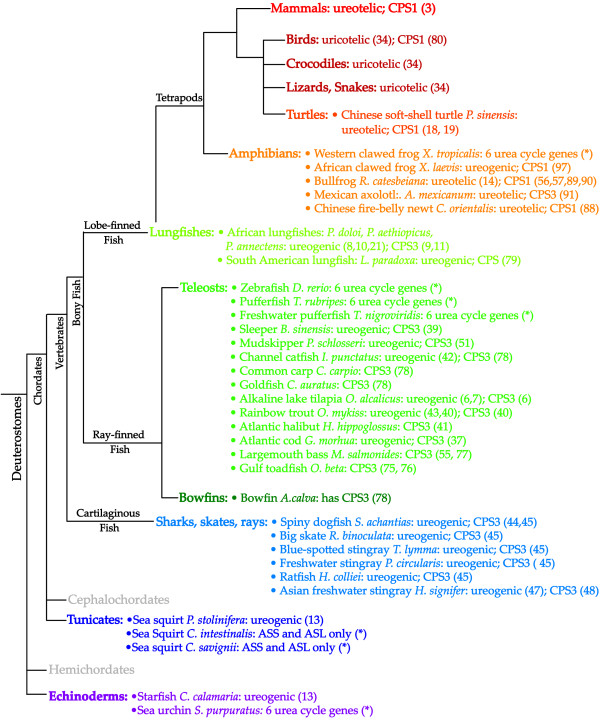
**Distribution of the ability to synthesize urea, CPSI and CPSIII in Deuterostomes.** Animals whose genomes were surveyed in this study are indicated with asterisks. All six urea cycle genes were identified in the genomes of zebrafish, pufferfish, freshwater pufferfish and sea urchin, indicating potential ability of these animals to synthesize urea. Full sets of urea cycle genes were not found in the genomes of sea squirts *C. intestinalis *and *C. savygnii*. Numbers in parentheses are numbers in the reference list. The cladogram indicates taxonomic relationships among phyla; the length of each clade does not indicate evolutionary distance between phyla.

Sequenced genomes of animals were surveyed for the presence of all six urea cycle genes. Human urea cycle genes were used as queries in BLAST searches of genomes from chicken (*Gallus gallus*), African clawed frog (*Xenopus laevis*), western clawed frog (*Xenopus tropicalis*), zebrafish (*Danio rerio*), pufferfish (*Takifugu rubripes*), freshwater pufferfish (*Tetraodon nigroviridis*), sea urchin (*Strongylocentrotus purputratus*), sea squirts (*Ciona intestinalis *and *Ciona savignyi*), fruit flies (*Drosophila melanogaster *and *Drosophila pseudoobscura*), honeybee (*Apis mellifera*), mosquito (*Anopheles gambiae*) and nematodes (*Caenorhabditis elegans *and *Caenorhabditis briggasae*). The presence of all six urea cycle gene in a genome would indicate that organism's potential to synthesize urea from ammonia. Figure 4 shows the cladogram of Deuterostomes in which the ureogenic ability of each organism is indicated, together with the type of CPS in organisms that were previously studied: a starfish (*Coscinasterias calamaria*), sea squirt (*Pyura stolonifera*) and several species of fish [6,8,10,11,13,21,37,38,40-43,51,55,75-79]. Urea cycle genes, including NAGS, were identified in frogs, all three teleost fish and sea urchin, an invertebrate. Although homologs of four urea cycle genes (CPSI, ornithine transcarbamylase, argininosuccinate synthase and argininosuccinate lyase) were previously identified in the chicken [80,81]; sequences similar to human NAGS and arginase I were absent from the chicken genome. This is consistent either with absence of NAGS and arginase I genes from the chicken genome, or presence of these two genes in the regions of genomes that have not been fully assembled at the time of our survey. Only argininosuccinate synthase and argininosuccinate lyase were found in sea squirts (Figure 4 and Additional File 1); however, a different species of sea squirt, *P. stolonifera*, has been reported to be able to synthesize urea [13]. The results of the survey of the insect and nematode genomes are listed in Additional File 1. Figure 4 also indicates that invertebrates and fish have CPSIII, while tetrapods have CPSI, suggesting that the transition from CPSIII to CPSI coincided with the conquest of land by tetrapods, as noted previously [53].

### Effect of Arginine on Enzymatic Activity of NAGS from Different Organisms

**Figure 5:**
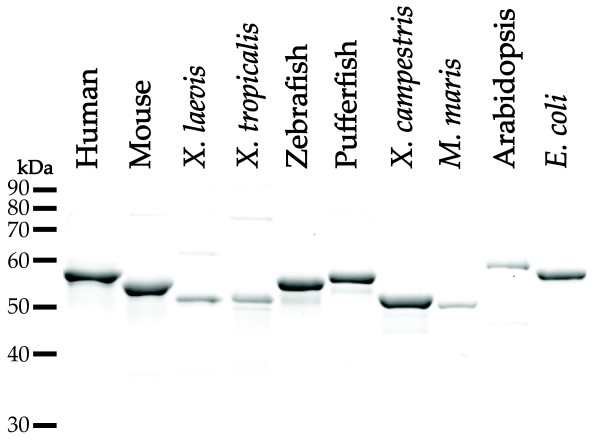
**Purification of recombinant NAGS from vertebrates, plant and bacteria.** Each protein had N-terminal polyhistidine affinity tag, was overexpressed in *E. coli *and purified using nickel-affinity chromatography.

The enzymatic activity of mammalian NAGS increases in the presence of arginine [68-71], while microbial and plant NAGS are inhibited by arginine [4,58]. To examine when the effect of arginine changed from inhibition to activation, we cloned and purified NAGS from *X. laevis*, *X. tropicalis*, zebrafish, pufferfish, an alpha-proteobacterium *M. maris *and arabidopsis, and examined their activity in the presence and absence of 1 mM arginine. Human and mouse NAGS were used as controls for enzyme activation, and *E. coli *NAGS and *X. campestris *NAGS-K were used as controls for NAGS inhibition. Bacterial expression plasmids containing human, mouse, *X. laevis*, *X. tropicalis*, zebrafish, pufferfish, *X. campestris*, *M. maris*, arabidopsis and *E. coli *NAGS were transformed into *E. coli *cells, and the corresponding recombinant proteins were overexpressed and purified using nickel-affinity chromatography. Figure 5 shows that each recombinant protein was purified to apparent homogeneity.

Table 3 shows the effects of the addition of 1 mM arginine on the enzymatic activities of vertebrate, bacterial and plant NAGS. The enzymatic activities of human, mouse and frog NAGS increased in the presence of 1 mM arginine. While frog NAGS had about 100-fold lower specific activities than other recombinant proteins, in the presence of 1 mM arginine, enzymatic activities of NAGS from *X. laevis *and *X. tropicalis *increased 20% and 38%, respectively. Enzymatic activities of NAGS from zebrafish and pufferfish were inhibited approximately 50% and 20% respectively, in the presence of arginine. Increasing the arginine concentration to 5 mM did not result in further inhibition of zebrafish and pufferfish NAGS (data not shown). As expected, NAGS from Arabidopsis, *M. maris*, *X. campestris *and *E. coli *were inhibited by 1 mM arginine. Table 3 shows that NAGS from *E. coli *was partially inhibited by 1 mM arginine. It was reported previously that NAGS from *E. coli *enzyme had 15% residual enzymatic activity in the presence of 1 mM arginine [82]. The difference between this study and our results may be due to differences in assay conditions and enzyme preparation (partially purified vs. recombinant enzyme). Inhibition of *X. campestris *NAGS-K and *E. coli *NAGS by arginine, as well as activation of mammalian NAGS by arginine, have been shown previously [62,68,69,82,83]. These results indicate that the inversion of the arginine effect on NAGS enzymatic activity occurred in amphibians and coincided with the transition from CPSIII to CPSI and evolution of tetrapods.

**Table 3:** Effects of L-arginine on vertebrate, plant and bacterial NAGS.

	**NAGS Specific Activity ** (μmoles min^-1^mg^-1^)	
	
**Organism**		**+ 1 mM Arg**
Human	19.293 ± 0.384^a^	29.285 ± 1.211
Mouse	29.523 ± 1.076	46.541 ± 5.602
African clawed frog	0.438 ± 0.011	0.528 ± 0.004
Western clawed frog	0.098 ± 0.002	0.136 ± 0.005
Zebrafish	10.638 ± 0.090	5.475 ± 0.121
Pufferfish	20.887 ± 0.278	15.898 ± 0.141
*X. campestris*	111.741 ± 1.669	nd^b^
*M. maris*	62.004 ± 3.751	0.572 ± 0.136
Arabidopsis	9.116 ± 0.279	nd
*E. coli*	5.859 ± 0.220	1.840 ± 0.032

^a^Activities are averages of three measurements and the associated standard errors.

^b^Not detectable.

## Discussion

This study revealed that allosteric inversion of the arginine effect on enzymatic activity of NAGS occurred in amphibians. This change in biochemical properties of NAGS coincided with the transition from CPSIII to CPSI and the conquest of land by amphibians and mammals. We have shown that mouse NAGS and bacterial XcNAGS-K, both members of vertebrate-like NAGS family, share a common arginine binding site.

The arginine binding site is conserved in NAGS that are inhibited, as well as in those activated by this allosteric cofactor. We used mutagenesis to show that replacement of these conserved amino acids with the corresponding residues from *E. coli *NAGS and NAGK, which do not respond to arginine [73,74], abolishes inhibition of XcNAGS-K and activation of mouse NAGS by arginine. Therefore, the switch from inhibition to activation of NAGS by arginine resulted from the differences in the type of conformational change or altered protein dynamics induced by the binding of arginine.

This difference in type of conformational change may have been the result of a single amino acid replacement, similar to allosteric inversions of the ATP effect on the enzymatic activity of aspartate transcarbamylase [84], and effect of N-acetylglucosamine-6-phosphate on the glucosamine-6-phosphate deaminase [85]. However, it is more likely that it involved multiple amino acid replacements because of the gradual transition from complete inhibition of bacterial NAGS to partial inhibition of fish NAGS to 204–0% activation of frog NAGS to doubling of enzymatic activity of mouse NAGS. This may explain the lower conservation of NAGS across phyla compared to other urea cycle enzymes [59-62]. Inversion of the allosteric effect has been shown to be triggered by temperature change in some enzymes, revealing the thermodynamic factors that govern ligand binding and ensuing conformational change [86]. Examination of the temperature dependence of the allosteric effect of arginine on NAGS from different organisms could yield insights into the nature of conformation change that results from arginine binding. In addition to this, differences in the three-dimensional structures of bacterial and mammalian NAGS liganded with arginine will also reveal differences in the mechanisms of allosteric inhibition and activation of NAGS by arginine.

Eight of the ten NAGS proteins that were examined in this study belong to the vertebrate-like family of NAGS [62], and their response to arginine ranged from complete inhibition (*X. campestris *NAGS-K, *M. maris *NAGS) to partial inhibition (zebrafish and pufferfish NAGS) to activation (amphibian and mammalian NAGS) (Table 3). Our sequence alignment of NAGS from 25 organisms revealed only nine invariant amino acids (Figure 2). Five invariant amino acids are part of the NAGS hydrophobic core, as they are all buried in the interior of *Nisseria gonorrhoeae *NAGS [66]. Four invariant amino acids are responsible for arginine binding. They are also conserved in the structurally related NAGK from *T. maritima *[62] and play a functional role in binding of arginine to NAGS from *P. aeruginosa *[67]. Interestingly, none of the NAGS residues known to bind substrates or catalyze NAG formation [66] are conserved among the analyzed proteins.

Our survey of sequenced animal genomes and literature revealed that the ability to produce urea is widely spread in the animal kingdom (Figure 4 and Additional File 1). For example, we identified a full set of urea cycle genes in the sea urchin (Figure 4), and previous reports indicate that other invertebrates, such as flatworms, annelids, gastropods and bivalves, also have the ability to convert ammonia into urea [12,13,16,17,20,24-33]. Other invertebrates, like nematodes and arthropods, do not use urea cycle for nitrogen waste disposal [35] (Additional File 1). We found homologs of argininosuccinate synthase and argininosuccinate lyase and no other urea cycle genes in the genomes of sea squirts *C. intestinalis *and *C. savignyi*, suggesting that these two aquatic organisms lost their ability to produce urea. However, another sea squirt, *P. stolonifera*, can produce urea [13], which may be advantageous for the survival of this organism when exposed to air during low tides. The capacity of many invertebrates to synthesize urea, and the absence of a complete set of urea cycle genes in nematodes, arthropods, and some sea squirts is consistent with the presence of a urea cycle in the common ancestor of animals and the subsequent loss of ability to synthesize urea in some phyla.

All six urea cycle genes were identified in the genomes of zebrafish, pufferfish and freshwater pufferfish. There are several fish species for which the need for a urea cycle can be explained. Lungfish are periodically exposed to air and use the urea cycle to dispose of ammonia [8,9,11,79]. Elasmobranches use urea as osmolyte [38,45,47-50], and alkaline lake tilapia lives in lakes containing high concentrations of ammonia [6,7]. However, the function of urea cycle in zebrafish, pufferfish and other teleosts that are not exposed to these types of extreme environments is not clear. In these fish, the urea cycle may be important for embryonic development as developing rainbow trout (*Oncorhynchus mykiss*), Atlantic cod (*Gadus morhua*) and Atlantic halibut (*Hippoglossus hippoglossus*) all express CPSIII, ornithine transcarbamylase and arginase I during embryogenesis [37,40,41,43].

In amphibians, we identified all six urea cycle genes in the genomes of African and western clawed frogs, *X. laevis *and *X. tropicalis *(Figure 4 and Additional File 1). *X. laevis *is mostly aquatic and ammonotelic, but can produce urea upon prolonged exposure to air [87], while the type of main nitrogenous waste for *X. tropicalis *is not known. Other adult amphibians, such as bullfrog (*Rana catesbeiana*) and Chinese fire-belly newt (*Cynops orientalis*) are ureotelic and they have CPSI, which uses ammonia as a substrate [56,57,88-90]. The CPSIII of ureotelic Mexican axolotl *Ambystoma mexicanum*, can use both glutamine and ammonia as substrates, and the latter is the preferred substrate of this enzyme [91]. Urea cycle genes are also present in genomes of birds and reptiles which are uricotelic. Four urea cycle genes were found in the genome of chicken [80], and the ornithine transcarbamylase gene has been identified in, and cloned from, the lizard *Sceloporus undulatum*, alligator *Alligator missippiensis *and ball python *Python regius *(Additional File 1). Our BLAST query of the chicken genome with human NAGS sequence yielded no results, which is consistent with the requirement for dietary arginine in chickens [92,93]. Two urea cycle genes, argininosuccinate synthase and lyase, have been retained in the chicken because they also function in nitric oxide signaling [94]. However, the presence of OTC and CPSI genes in the chicken genome, as well as their sporadic expression in various organs [80,81] appear to be vestigial remnants from the tetrapod common ancestor, that we believe was ureogenic.

Our survey of literature and sequence databases indicates that all tetrapods have CPSI, whereas all other animals, aquatic and terrestrial, if ureogenic, have the CPSIII gene (Figure 4 and Additional File 1). NAG is an essential allosteric activator of CPSI in mammals and frogs [3,90]. NAG also activates CPSIII from Atlantic halibut, spiny dogfish, and largemouth bass [41,44,55], while CPSIII from alkaline lake tilapia does not require this cofactor for function [6]. Because NAG is important for enzymatic activity of both CPSI and CPSIII we wanted to examine when during evolution the allosteric inversion of arginine effect on NAGS activity occurred. Our results indicate that the allosteric effect of arginine on NAGS activity changed during evolution of amphibians, from partial inhibition in zebrafish and pufferfish to activation in African and western clawed frogs (Table 3). Partial inhibition of zebrafish and pufferfish NAGS by arginine was surprising since NAG is expected to be an activator of CPSIII in these organisms, similar to CPSIII from Atlantic halibut, spiny dogfish and largemouth bass. However, under conditions of elevated glutamine between 10 and 15 mM, the CPSIII in these latter fish become less dependent on NAG [41,44,55]. The concentration of glutamine in fish mitochondria is not known, but in the rat liver mitochondria it is between 6.6 and 15 mM [95,96]. Assuming that the metabolite concentrations in fish and mammalian mitochondria are similar, fish CPSIII would then be expected to be saturated with glutamine, and NAG may be playing a role in stabilization of CPSIII instead of its activation.

Fish CPSIII can also use ammonia as a substrate *in vitro*, and NAG is essential for CPSIII activity under those conditions [41,44,55]. The transition between glutamine-dependent CPSIII and ammonia-dependent CPSI occurred in amphibians. CPSI from *R. catesbeiana *cannot bind glutamine, and CPSI from *X. laevis *cannot catalyze the glutaminase reaction due to a mutation of a cysteine located in the active site of glutaminase domain [56,57,97]. Our results indicated that NAGS from *X. laevis *and *X. tropicalis *were both activated by arginine (Table 3). Enzymatic activities of both frog enzymes were also about one hundred-fold lower than enzymatic activities of other enzymes we studied. This may have been due to either lack of selective pressure to maintain high enzymatic activity in mostly aquatic and ammonotelic frogs [87] or presently unknown assay requirements. We expect that NAGS activity of ureotelic bullfrog *R. catesbeiana *may be higher; unfortunately, the genome and expressed genes of this organism are not being sequenced, precluding ready cloning and examination of the enzymatic activity and response to arginine of bullfrog NAGS. Taken together, these results indicate that the switch from arginine inhibition to arginine activation of NAGS occurred in amphibians, and was concurrent with the transition from CPSIII to CPSI and the tetrapod conquest of land.

CPSI is the rate limiting enzyme of the mammalian urea cycle because all CP produced by this enzyme is converted into urea, and under normal physiological conditions none of the urea cycle intermediates accumulate [98]. Because NAG is essential for the activity of CPSI, and arginine activates mammalian NAGS, it has been proposed that arginine may play a role in regulation of ureagenesis [99-105]. Arginine is also an intermediate of urea cycle [3] and the amino acid with the largest number of nitrogen atoms. Assuming that changes of intramitochondrial concentration of arginine indeed modulate enzymatic activity of NAGS, the activation of NAGS by arginine in conjunction with the activation of CPSI by NAG form a double positive feedback loop. This is similar to double positive feedback loops found in calcium signaling cascades, polarization in budding yeast and cell-cycle control in *Xenopus *oocytes, which are responsible for rapid and robust regulation of signaling pathways [106]. This double positive feedback system appears to be absent in fish, where NAGS is partially inhibited by arginine and the enzymatic activity of CPSIII is not absolutely dependent on NAG. Therefore, while the fish urea cycle may be capable of efficient conversion of ammonia into urea, because direct ammonia clearance can occur, it does not have to be as robust and sensitive to changes in the nitrogen load as the mammalian urea cycle. CPSI appears to have evolved from CPSIII following the loss of its ability to bind glutamine [56,57,97]. The resulting CPSI became completely dependent on NAG, and together with the inversion of the arginine effect on NAGS activity, have resulted in a robust detoxification system that is more efficient at protecting the central nervous system from the harmful effects of ammonia and provided a decisive selective advantage for the tetrapod terrestrial expansion.

## Conclusion

The arginine binding site is conserved in vertebrate-like NAGS, which are inhibited by arginine in bacteria and fish, and activated by this cofactor in amphibians and mammals. Therefore, the differences in allosteric effect of arginine on NAGS result from different conformational changes induced by binding of arginine to NAGS. The change in arginine allosteric effect on NAGS coincided with terrestrial conquest by tetrapods.

## Methods

### Site-directed Mutagenesis

Arginine-insensitive mutants of mouse NAGS and *X. campestris *NAGS-K were generated using mutagenesis primers listed in Table 1 of the Additional File 2, plasmids pTOPOmNAGS-M [69] and pTOPOXcNAGS-K [62] as templates, and the QuickChange Site-Directed Mutagenesis kit (Stratagene) according to the manufacturer's instructions. The presence of each mutation was confirmed by sequencing. Plasmids harboring coding sequences of mutant mNAGS and XcNAGS-K were cleaved with *Nde*I and *Bam*HI and inserts were subcloned into pET15b expression plasmids.

### Protein Expression and Purification

Expression plasmids were transformed into C41(DE3) *E. coli *cells and overexpression of recombinant proteins was induced using an Overnight Expression Autoinduction System 1 (Novagen). Recombinant proteins were purified with HandeeSpin columns (Pierce), as described previously [69]. Plasmids pET15bmNAGS-M [69], pET15bXcNAGS-K [62] and pET15bEcNAGS (generously provided by Dr. Dashuang Shi, Research Center for Genetic Medicine, Children's National Medical Center) were used for overexpression and purification of recombinant mouse NAGS, *X. campestris *NAGS-K and *E. coli *NAGS, respectively. NAGS from *X. tropicalis *and *X. laevis *were purified as described previously [69], with some modifications. Recombinant NAGS from *X. laevis *and *X. tropicalis *were overexpressed in the Rosetta 2 cells (Novagen) using induction with 0.1 mM IPTG for 4 hours at 37°C, and 10 mM glutamate was added to all buffers used for cell lysis and protein purification to help stabilize the recombinant proteins throughout the purification procedure. Recombinant NAGS from *M. maris *was overexpressed in Rosetta 2 cells (Novagen) following overnight induction of expression with 0.2 mM IPTG at room temperature. *M. maris *NAGS was purified, as described previously [107].

Expression plasmids harboring wild-type and arginine-insensitive mouse and *X. campestris *NAGS-K were transformed into C41(DE3) *E. coli *cells and overexpression of recombinant proteins was induced using Overnight Expression Autoinduction System 1 (Novagen). Recombinant proteins were purified using HandeeSpin columns (Pierce), as described previously [69].

### Enzyme Assays

Enzymatic activities of purified proteins were measured, as described previously [108], with the exception of NAGS from *X. tropicalis *and *X laevis*. Enzymatic activities of frog NAGS were carried out at room temperature, and with 50 mM glutamate and 12.5 mM AcCoA, which is five-fold higher than our standard assay conditions [108].

### Identification of Urea Cycle Genes Across Phyla

Genomes of zebrafish (*D. rerio*), pufferfish (*T. rubripes*), freshwater pufferfish (*T. nigroviridis*), African clawed frog (*X. laevis*), Western clawed frog (*X. tropicalis*), sea urchin (*S. purpuratus*), sea squirts (*C. intestinalis *and *C. savygnii*), fruit flies (*D. melanogaster *and *D. pseudoobscura*), honeybee (*A. mellifera*), mosquito (*A. gambiae*) and roundworms (*C. elegans *and *C. briggasae*) were queried with the human NAGS, CPSI, ornithine transcarbamylase, argininosuccinate synthase, argininosuccinate lyase and arginase 1 sequences. When sequences similar to the query were not present, genomes were also queried with the *E. coli *NAGS, CPS, ornithine transcarbamylase, argininosuccinate synthetase, and argininosuccinate lyase. Urea cycle genes that were identified are listed in Table S1.

### Identification and Cloning of the NAGS Genes from Fish, Frogs, Plant and Bacteria

The plant NAGS genes were identified based on their similarity with NAGS from *E. coli *[62]. The expressed sequence tag (EST) database was queried with the predicted *Arabidopsis *NAGS protein to identify clones that contained full length cDNA for NAGS genes from plants. A plasmid containing the full length sequence for the *Arabidopsis *NAGS (Accession# U20510) was identified and obtained from The Arabidopsis Information Resource. This plasmid was used as a template for amplification of the coding sequence with primers 1 and 2 (Table 2 in Additional File 2). The amplification conditions were: 3 min. initial denaturation at 95°C followed by 25 cycles of 30 s denaturation at 95°C, 30 s annealing at 55°C and 1.5 min. extension at 72°C, and 5 min. final extension at 72°C using *Pfu *Turbo Hotstart DNA polymerase (Stratagene). The amplification product was subcloned into pCR4Blunt-TOPO plasmid (Invitrogen) producing pTOPOaNAGS. The correct amino acid sequence was confirmed by sequencing. pTOPOaNAGS was cleaved with *Ase*I and *Bam*HI and *Arabidopsis *NAGS was cloned into pET15b expression plasmid (Novagen), cleaved with *Nde*I and *Bam*HI. The *Ase*I restriction endonuclease was used because an *Nde*I site is present in the *Arabidopsis *NAGS coding sequence and the two enzymes have compatible cohesive ends. The resulting plasmid, called pET15baNAGS, allows overexpression of the recombinant protein with the N-terminal polyhistidine affinity tag.

Zebrafish (*Danio rerio*) and pufferfish (*Takifugu rubripes*) NAGS genes were identified based on their similarity with mammalian NAGS [62]. The EST database was queried with the predicted pufferfish NAGS protein sequence and two plasmids containing full length sequence of the pufferfish NAGS were identified (Accession# CA846082 and BU808158) and obtained from ATCC. The coding sequence of pufferfish NAGS was amplified using EST clones as template, primers 3 and 4 (Table 2 in Additional File 2) and the same amplification conditions as for *Arabidopsis *NAGS coding sequence. The amplification product was subcloned into pCR4Blunt-TOPO plasmid (Invitrogen) and sequenced to confirm that it codes for the correct amino acid sequence. The coding sequence for pufferfish NAGS was excised with *Nde*I and *Bam*HI from the TOPO-plasmid and cloned into pET15b plasmid resulting in pET15bfNAGS-M.

A query of the EST database with the predicted zebrafish NAGS protein did not identify a plasmid containing the full length coding sequence for zebrafish NAGS. A cDNA library prepared form adult zebrafish mRNA and inserted into pExpress-1 plasmid was obtained from Open Biosystems (cat# LDR1216) and used to clone zebrafish NAGS coding sequence. The first round of amplification was done with primers 5 and 6 (Table 2 in Additional File 2) and 100 ng of plasmid cDNA library as the template and the following conditions: 3 min. initial denaturation at 95°C, followed by 30 cycles of 30 s denaturation at 95°C, 30 s annealing at 55°C and 1.5 min extension at 72°C, and 5 min. final extension at 72°C. The second round of amplification was carried out with primers 7 and 8 (Table 2 in Additional File 2), 1 μl of amplification product from the first round and the following conditions: 3 min. initial denaturation at 95°C, followed by 20 cycles of 30 s denaturation at 95°C, 30 s annealing at 55°C and 1.5 min extension at 72°C, and 5 min. final extension at 72°C. The amplification product was subcloned into pCR4Blunt-TOPO plasmid (Invitrogen) and sequenced to confirm that it codes for the correct amino acid sequence. The coding sequence for zebrafish NAGS was subcloned into pET15b resulting in pET15bzfNAGS-M expression plasmid.

A query of the EST database with predicted protein sequences of NAGS from *X. laevis *and *X. tropicalis *identified two plasmids containing their full length coding sequences. Plasmid containing the coding sequence of *X. tropicalis *NAGS was obtained from ATCC (cat # 5592351) and plasmid with *X. laevis *NAGS coding sequence was obtained from Open Biosystems (cat# EXL1051-5611590). The coding sequence of *X. laevis *NAGS was amplified using the EST clone as a template, primers 9 and 10 (Table 2 in Additional File 2) with the same amplification conditions used for the *Arabidopsis *NAGS coding sequence. The amplification product was subcloned into pCR4Blunt-TOPO plasmid (Invitrogen) producing pTOPOXlNAGS-M. The correct amino acid sequence was confirmed by sequencing. The pTOPOXlNAGS was cleaved with *Ase*I and *Bam*HI and *X. laevis *NAGS was cloned into pET15b expression plasmid (Novagen), cleaved with *Nde*I and *Bam*HI. The *Ase*I restriction endonuclease was used because an *Nde*I site is present in the *X. laevis *NAGS coding sequence and the two enzymes have compatible cohesive ends. The resulting plasmid was called pET15bXlNAGS-M. The coding sequence of *X. tropicalis *NAGS contained *Nde*I and *BamH*I restriction sites. Site directed mutagenesis was used to introduce silent mutations that remove *Nde*I and *BamH*I restriction sites without affecting the amino acid sequence. Plasmid with this modified coding sequence of *X. tropicalis *NAGS was used as a template for amplification with primers 11 and 12 (Table 2 in Additional File 2) and the same conditions as described for *Arabidopsis *NAGS. The amplification product was subcloned into pCR4Blunt-TOPO plasmid (Invitrogen) and sequenced to confirm that it codes for the correct amino acid sequence. The coding sequence for *X. tropicalis *NAGS was subcloned into pET15b, resulting in expression plasmid pET15bXtNAGS-M.

The coding sequence of *M. maris *NAGS gene was amplified from *M. maris *MCS10 genomic DNA (generously provided by Dr. Craig Stephens, Biology Department, Santa Clara University, 500 El Camino Real, Santa Clara, CA) using Phusion™ polymerase (Finnzymes, New England BioLabs) and the primers 13 and 14 (Table 2 in Additional File 2). Amplification conditions were: 30 s at 98°C for the polymerase activation and 40 cycles of denaturation for 15 sec. at 98°C, annealing for 15 sec. at 60°C and extension for 30 sec. at 72°C, followed by final extension for 10 min. at 72°C, yielding a unique fragment of the desired size. The PCR product was cloned into a TOPO vector using a Zero-Blunt TOPO cloning kit (Invitrogen). *Nde*I and *Xho*I were used to move the *M. maris *NAGS coding sequence from the TOPO plasmid into bacterial expression vector pET28a (Novagen), resulting in the plasmid pET28aMmNAGS.

## Abbreviations

CP: carbamylphosphate; CPSI: carbamylphosphate synthetase I; CPSIII: carbamylphosphate synthetase III; mNAGS: mouse N-acetylglutamate synthase; NAG: N-acetylglutamate; NAGK: N-acetylglutamate kinase; NAGS: N-acetylglutamate synthase; NAGS-K: bifunctional N-acetylglutamate synthase-kinase; XcNAGS-K: bifunctional N-acetylglutamate synthase-kinase from *X. campestris*

## Authors' contributions

NH carried out cloning, purification and measurements of enzymatic activity of frog NAGS. MP carried out cloning, purification and measurements of enzymatic activity of pufferfish, arabidopsis and X. campestris NAGS. QQ performed directed mutagenesis of mNAGS and XcNAGS-K, purification of wild-type and mutant proteins and measurements of XcNAGS-K kinase activity. HM carried out cloning, purification and measurements of enzymatic activity of zebrafish NAGS. JC-L performed cloning, purification and measurements of enzymatic activity of NAGS from *M. maris*. HM and MT have been involved in critical revisions of the manuscript. LC conceived the study, carried out measurements of enzymatic activities of mutant and wild-type mNAGS and XcNAGS-K and drafted the manuscript. All authors read and approved the final manuscript.

## Supplementary Material

Additional file 1 Urea cycle genes in animals. Genomes of animals that belong to different phyla were surveyed for the presence of urea cycle genes. Accession numbers and databases that were searched are listed for each urea cycle gene. Click here for file

Additional file 2 Primers used for site-directed mutagenesis and cloning of NAGS. Table 1 lists primers that were used for site-directed mutagenesis of mouse NAGS and *X. campestris *NAGS-K. Table 2 lists primers that were used for cloning of the arabidopsis, pufferfish, zebrafish, *X. laevis*, *X. tropicalis *and *M. maris *NAGS, and sizes of amplification products for each primer pair. Click here for file
