# Supplementary material for: Inversion of allosteric effect of arginine on N-acetylglutamate synthase, a molecular marker for evolution of tetrapods
Source: BMC Biochem. 2008 Sep 18;9:24. doi: 10.1186/1471-2091-9-24 (PMC2566978; doi:10.1186/1471-2091-9-24)
Supplement: Additional file 2 — Primers used for site-directed mutagenesis and cloning of NAGS. Table 1 lists primers that were used for site-directed mutagenesis of mouse NAGS and X. campestris NAGS-K. Table 2 lists primers that were used for cloning of the arabidopsis, pufferfish, zebrafish, X. laevis, X. tropicalis and M. maris NAGS, and sizes of amplification products for each primer pair. [file 1471-2091-9-24-S2.doc]

**Table 1.** Mutagenic primers used to introduce amino acid changes which abolish binding of arginine by mouse NAGS and *X. campestris* NAGS-K.

| **Gene** | **Mutation** | **Primer** |
| --- | --- | --- |
| Mouse NAGS | F121C | 5’-CACTGGCTCACGCAATGCCAGACATGTTATCATTCGGTGGAC-3’ |
|  |  | 5’-GTCCACCGAATGATAACATGTCTGGCATTGCGTGAGCCAGTG-3’ |
|  | E354A | 5’-ACGCTGCTCACGGCACTCTTTAGTAACAAGGGC-3’ |
|  |  | 5’-GCCCTTGTTACTAAAGAGTGCCGTGAGCAGCGT-3’ |
|  | G360P | 5’-CTCTTTAGTAACAAGCCGTGTGGTACCCTGTTTAAAAATGCTGAG-3’ |
|  |  | 5’-CTCAGCATTTTTAAACAGGGTACCACACGGCTTGTTACTAAAGAG-3’ |
|  | G362S | 5’-AGTAACAAGGGCTGTTCCACCCTGTTCAAAAATGCTGAG-3’ |
|  |  | 5’-GCCCTTGTTACTAAAGAGTGCCGTGAGCAGCGT-3’ |
| *X. campestris* NAGS-K | F35C | 5’-GAGATCAGCCAGTATTTAAAGCGTTGCTCGCAGCTGGAC-3’ |
|  |  | 5’-GTCCAGCTGCGACGAACGCTTTAAATACTGGCTGATCTC-3’ |
|  | E280A | 5’-CCGGCGGATCTGGCTAAGGCTCTGTTCACCCAC-3’ |
|  |  | 5’-GTGGGTGAACAGAGCCTTAGCCAGATCCGCCGG-3’ |
|  | G286P | 5’-CTGTTCACCCACAAGCCGTCCGGTACCTTGGTGCGGCGTGGC-3’ |
|  |  | 5’-GCCACGCCGCACCAAGGTACCGGACGGCTTGTGGGTGAACAG-3’ |
|  | G288S | 5’-ACCCACAAGGGTTCCTCCACGTTGGTACGTAGAGGCGAGCGC-3’ |
|  |  | 5’-GCGCTCGCCTCTACGTACCAACGTGGAGGAACCCTTGTGGGT-3’ |

**Table 2.** Primers used for amplification of the coding sequences of NAGS from arabidopsis, pufferfish, zebrafish, *X. laevis*, *X. tropicalis* and *M. maris*.

| **Primer** | **Sequence** | **Amplicon (size)** |
| --- | --- | --- |
| 1 | 5'-AGATTAATGTCGGCGATAAGCAATTTGTGC-3' | Arabidopsis NAGS coding sequence (1638 bp) |
| 2 | 5'-TTGGATCCTTATTATCAAGAATCATACTGAAATGTTCG-3' |
| 3 | 5'-TTCCATATGGCGAAACTCAACAGCGGCTCC-3' | Pufferfish NAGS coding sequence(1513 bp) |
| 4 | 5'-CCGGATCCTTATTATCAGGATTTTGCAGCAGCTGTGAG-3' |
| 5 | 5’-TCCAACTCCTCAGGTCAACGAAAGCTA-3’ | Zebrafish NAGS coding sequence (1710 bp) |
| 6 | 5’-GGTGAGTAAGTGATTGTGTGAACCATCCCC-3’ |
| 7 | 5'-CCTCTGCGACATATGGCCAAAGTCAACAGTGGTTCG-3' | Zebrafish NAGS coding sequence (1468 bp) |
| 8 | 5'-GAGAGGATCCTTATTATTATGAGCCGTGGTGCTGCTGAAGAGG-3' |
| 9 | 5’-GCAAGAATTAATGCTCGGCCTGCCCACG-3’ | *X. laevis* NAGS coding sequence (1401 bp) |
| 10 | 5’-CCTGGATCCTTATTATCATGATATTTGAGGTTTGCAGAAGG-3’ |
| 11 | 5’-CAGAACTTACATATGGCAATAGTGAAGGGCTTTTCTACC-3’ | *X. tropicalis* NAGS coding sequence (1503 bp) |
| 12 | 5’-CCTGGATCCTTATTATCATGAAACCTGAGGCTTGCAGAAGG-3’ |
| 13 | 5’-CATATGAATCCGAATGCACCGGG-3’ | *M. maris* NAGS-K coding sequence (1335 bp) |
| 14 | 5’-GGATCCTCATTGCGGCGCCTCAAGGGT-3’ |
